# Overexpression of MMP-7 increases collagen 1A2 in the aging kidney

**DOI:** 10.1002/phy2.90

**Published:** 2013-10-11

**Authors:** Anna Ślusarz, LaNita A Nichols, Elizabeth A Grunz-Borgmann, Gang Chen, Adebayo D Akintola, Jeffery M Catania, Robert C Burghardt, Jerome P Trzeciakowski, Alan R Parrish

**Affiliations:** 1 Medical Pharmacology and Physiology, School of Medicine, University of MissouriColumbia, Missouri; 2 Department of Systems Biology & Translational Medicine, College of Medicine, Texas A&M University System Health Science Center; 3 Department of Veterinary Integrated Biosciences, College of Veterinary Medicine, Texas A&M University

**Keywords:** Aging, collagen, fibrosis, MMP-7

## Abstract

The percentage of the U.S. population over 65 is rapidly increasing, as is the incidence of chronic kidney disease (CKD). The kidney is susceptible to age-dependent alterations in structure, specifically tubulointerstitial fibrosis that leads to CKD. Matrix metalloproteinases (MMPs) were initially characterized as extracellular matrix (ECM) proteinases; however, it is clear that their biological role is much larger. We have observed increased gene expression of several MMPs in the aging kidney, including MMP-7. MMP-7 overexpression was observed starting at 16 months, with over a 500-fold upregulation in 2-year-old animals. Overexpression of MMP-7 is not observed in age-matched, calorically restricted controls that do not develop fibrosis and renal dysfunction, suggesting a role in the pathogenesis. In order to delineate the contributions of MMP-7 to renal dysfunction, we overexpressed MMP-7 in NRK-52E cells. High-throughput sequencing of the cells revealed that two collagen genes, *Col1a2* and *Col3a1*, were elevated in the MMP-7 overexpressing cells. These two collagen genes were also elevated in aging rat kidneys and temporally correlated with increased MMP-7 expression. Addition of exogenous MMP-7, or conditioned media from MMP-7 overexpressing cells also increased Col1A2 expression. Inhibition of protein kinase A (PKA), src, and MAPK signaling at p38 and ERK was able to attenuate the MMP-7 upregulation of Col1a2. Consistent with this finding, increased phosphorylation of PKA, src, and ERK was seen in MMP-7 overexpressing cells and upon exogenous MMP-7 treatment of NRK-52E cells. These data suggest a novel mechanism by which MMP-7 contributes to the development of fibrosis leading to CKD.

## Introduction

More than 10% of the adult population in the United States suffers from chronic kidney disease (CKD) (Levey and Coresh 2012), and the prevalence increases with age with more than 35% of those over 60 affected. CKD is associated with various disease states, primarily old age, diabetes, hypertension, obesity, and cardiovascular disease, but can also result from infections and exposure to drugs or toxins. In the early stage, CKD is mostly asymptomatic, although associated with risk of cardiovascular morbidity and mortality. As kidney function deteriorates through more extensive damage to the organ it becomes impossible to reverse the progression to end-stage kidney failure, which is defined by glomerular filtration rate (GFR) of less than 15 mL/min per 1.73 m^2^. Complications of such low GFR include an increased risk of cardiovascular disease, acute kidney injury, infection, cognitive impairment, and impaired physical function (Levey and Coresh 2012), and require intervention in the form of dialysis or kidney transplantation. It is thus critical to find targets for intervention in the progression of CKD to end-stage kidney failure.

Collagens are extracellular matrix (ECM) proteins, which play a role in organ formation, growth, and homeostasis. Fibrosis results from abnormal accumulation of matrix, predominantly collagen, which is associated with loss of organ function as normal tissue is replaced by scar tissue (Wynn 2007). CKD is a prototypical example of progressive fibrosis leading to organ failure (Hewitson 2009; Boor et al. 2010; Zeisberg and Neilson 2010). Both glomerulosclerosis and tubulointerstitial fibrosis are involved in CKD, however, the latter is the better histological predictor of progression (Bohle et al. 1987). Increased expression of *Col1a2* and *Col3a1* have been previously described to correlate with aging, injury, and fibrotic changes in the kidney (Bielesz et al. 2010; Gaikwad et al. 2010; Fragiadaki et al. 2011), as well as in other systems (Wu and Chakravarti 2007; van Almen et al. 2011).

Numerous animal models have been described to study age-related alterations in the kidney (Baylis and Corman 1998). Many of the structural changes in the aged human kidney are observed in rats, such as degenerative changes in the proximal tubules and thickening of the glomerular basement membrane. Other notable functional deficits in the rat include proteinuria and reduced urine concentration (Haley and Bulger 1983; Sands 2003). Of note, the development of renal disease is more severe in males as compared to females (Baylis 1994; Sasser et al. 2012), and that nutrition affects age-related renal dysfunction (Zawada et al. 1997). In male Fischer 344 rats, we observe a progression of kidney deterioration similar to end-stage renal disease including severe glomerulosclerosis and interstitial fibrosis (Corman and Owen 1992). Lifelong caloric restriction will ameliorate this effect (Stern et al. 2001). Rat models present a well-characterized and invaluable tool to investigate age-related changes in the kidney, including consequences of glomerulosclerosis and fibrosis.

Given the development of glomerulosclerosis and tubulointerstitial fibrosis in the aging kidney, both of which are associated with increased ECM deposition, it was suggested that MMP activity would decrease during aging. In aging male Wistar kidneys, proximal tubules have been shown to have lower cysteine and metalloproteinase activity (Schaefer et al. 1994); similar results were seen in brush border–enriched fractions of male Sprague–Dawley rats (Reckelhoff and Baylis 1992). In both studies, however, the activities of specific MMPs were not characterized. However, in a microarray analysis of kidney samples from 74 patients between 27 and 92 years indicated a 2.90-fold increase in MMP-7 expression with increasing age (Rodwell et al. 2004). Interestingly, the fold change was the second largest. This finding has been confirmed in a separate study (Melk et al. 2005). Previous studies from our laboratory have indicated that MMP-7 is overexpressed in the aging rat kidney (Chen et al. 2007).

MMP-7 is the smallest member of the metalloproteinase family and has gained attention in the recent years for its role in abnormal tissue remodeling (Nagase and Woessner 1999). The secreted protein is minimally expressed in the adult, with the notable exceptions of the small intestine and bladder. MMP-7 is not detected in normal human renal tubular epithelium, but significant expression was seen in a number of pathologic states including polycystic kidney disease in humans and unilateral ureteral obstruction or acute folic acid nephropathy in mice (Surendran et al. 2004). It has been proposed as a new screening marker for kidney damage (Reich et al. 2011), cardiovascular complications in patients with CKD (Musial and Zwolinska 2012), and possibly for the prediction of kidney transplant rejection (Jovanovic et al. 2008; Rodder et al. 2010). In addition, MMP-7 may be involved in the development of fibrosis in the lung (Zuo et al. 2002; Rosas et al. 2008) and liver (Huang et al. 2005). There have been reports of MMP inhibitors, specifically doxycycline, successfully reducing proteinuria in patients with diabetic nephropathy (Aggarwal et al. 2010) and glomerulonephritis (Ahuja 2003), suggesting that MMPs play a pathogenic role in the development of chronic renal dysfunction. In this study, we investigated the mechanistic link between MMP-7 overexpression and fibrosis in the aging kidney.

## Material and Methods

### Animals

Male Fisher 344 rats were obtained from the National Institute of Aging, Bethesda, MD, and housed in the Animal Facilities at the College of Medicine, Texas A&M Health Science Center or the University of Missouri School of Medicine. All animal protocols were submitted and approved by the Texas A&M and University of Missouri Animal Care and Use Committee in accordance with the NIH.

Animals were purchased at the indicated ages and housed for a week before being placed in metabolic cages (Tecniplast, Exton, PA) 18 h prior to sacrifice. Animals were fed ad libitum (AL) or calorie restricted (CR); CR was initiated at 14 weeks of age at 10% restriction, increased to 25% restriction at 15 weeks, and to 40% restriction at 16 weeks, which was subsequently maintained throughout the remaining life of the animal. The animal room was temperature controlled and maintained on a 12:12 h light:dark cycle. Following anesthesia (ketamine 87 mg/kg and xylazine 13 mg/kg body weight), rats were sacrificed by heart puncture, the abdominal cavity was opened, and the kidneys were removed and weighed. Kidneys were sliced into 1-mm-thick sections and either snap frozen in liquid nitrogen or frozen in liquid nitrogen–cooled optimal cutting temperature compound (Tissue-Tek; Sakura Finetek, Torrance, CA) for cryosectioning or fixed in formalin and paraffin embedded for immunohistochemistry.

### MMP-7 clones

The full-length wild-type human MMP7 (NM_002423) clone in pCMV6-Neo was purchased from OriGene (Rockville, MD). The sequence was altered by oligonucleotide-directed mutagenesis exchange reactions as described previously (Geiser et al. 2001) using QuickChange II Site-Directed Mutagenesis Kit (Stratagene/Agilent Technologies, Santa Clara, CA). The active mutant with a substitution of valine to glycine at amino acid 92 (Witty et al. 1994) was generated using the following oligonucleotides: antisense 5′- CAG ATG TGG AGG GCC AGA TGT TG-3′, and sense 5′- CAA CAT CTG GCC CTC CAC ATC TG-3′. The inactive mutant with a substitution of glutamic acid to glutamine at amino acid 216 was generated using the following oligonucleotides: antisense 5′- ATG GCC AAG TTG ATG AGT TGC-3′ and sense 5′- GCA ACT CAT CAA CTT GGC CAT-3′. Mutations were confirmed by sequencing.

### Cell culture

NRK-52E cells were obtained from the ATCC (catalog # CRL-1571; Manassas, VA) and maintained in DMEM/F12 1:1 (Dubelcco's modified, Eagle medium/Ham's F-12 Nutrient Mix; Gibco, Life Technologies, Grand Island, NY) supplemented with 5% FBS (fetal bovine serum; Hyclone, Thermo Fisher Scientific), penicillin/streptomycin, and gentamicin (Gibco, Life Technologies). The cells were transfected with the full-length human wild-type MMP7 (NM_002423), active and inactive mutants and control vector pCMV6-Neo (OriGene) using Lipofectamine 2000 (Invitogen, Life Technologies) and subjected to selection with 350 μg/mL Geneticin (Gibco, Life Technologies) in DMEM/F12 with 10% FBS and no other antibiotics. In certain experiments, conditioned medium was collected after 24 h and concentrated using Vivaspin columns with a molecular weight cut-off of 10 kDa (Sartorius, Bohemia, NY).

### Western blot

Subconfluent cells were washed twice with ice-cold PBS (phosphate buffered saline; Gibco, Life Technologies) and lysed with 10-mmol\L Tris-1% sodiumdodecyl sulphate (SDS) buffer with Halt Protease/Phosphatase inhibitor. Cells were scraped and incubated for 15 min at 4°C on a rocker. Cells were further disrupted by passing through a 20-gauge needle and spun at 12,000*g* for 15 min at 4°C. Tissue lysates were isolated using a 10-mmol\L Tris-1% SDS buffer supplemented with Halt Protease Inhibitor Cocktail (Thermo Fisher-Pierce, Rockford, IL). Protein concentration was determined by absorbance readings at 280 nm on a Nanodrop 2000c spectrophotometer (Thermo Fisher Scientific).

The following antibodies were used: anti-MMP7: GTX104658 1:1000 (GeneTex, Irvine, CA), anti-β actin A2228 1:2000 (Sigma, St. Louis, MO), ERK (4695), P-ERK (4370), src (2102), P-src (6943), protein kinase A (PKA) (4782), and P-PKA (4781) 1:1000 (all Cell Signaling Technology, Beverly, MA). Goat anti-rabbit horseradish peroxidase (HRP) conjugate and goat anti-mouse HRP conjugate (Jackson ImmunoResearch Laboratories, West Grove, PA) were used at 1:20,000 dilutions. Blots were developed using West Femto (Thermo Fisher-Pierce) and imaged using the ChemiDoc imaging system (Bio-Rad, Hercules, CA).

### Immunohistochemistry

Kidneys were sliced with a razor blade into four sagittal sections and placed in 4% paraformaldehyde for 24 h. The sections were subsequently rinsed repeatedly with PBS, and placed in 70% ethanol for embedding. Sections were deparaffinized by xylene incubation for 12 min and rehydrated in a graded series of ethanol (95%, 80%, 70%, and 50% ethanol) for 5 min each, and then washed with PBS for 10 min. Slides were stained for collagen deposition using the NovaUltra Sirius Red Stain Kit, IHC WORLD, Woodstock, MD.

### Immunofluorescence

NRK cells were grown on glass coverslips in 6-well plates. Cells were washed with PBS, fixed in 4% paraformaldehyde for 10 min, permeabilized with 1% Triton X-100 for 10 min, blocked with Background Sniper (Biocare Medical, Concord, CA) for 10 min, washed with tris buffered saline, and incubated with the following antibodies: MMP7 (SAB4501894, Sigma-Aldrich, St. Louis, MO; 1:100), src (2102, 1:100), P-src (6943, 1:100), ERK (4695, 1:100), P-ERK (4370, 1:200), PKA (4782, 1:100), and P-PKA (4781, 1:100) (Cell Signaling Technology) in 1% BSA (bovine serum albumin; Thermo Fisher Scientific) in PBS for 1 h at room temperature (RT). Negative control for secondary antibody was only incubated with Fluorescence Antibody Diluent (Biocare Medical). Coverslips were then washed with PBST (PBS with 0.2% Tween 20) and incubated with goat anti-rabbit secondary antibody DyLight 594 (Biocare Medical) 1:50 for 1 h at RT. Coverslips were then washed once and mounted on slides with Fluoroshield with 4′,6-diamidino-2-phenylindole (DAPI) (Sigma-Aldrich).

Cells were imaged on an Olympus IX51 microscope with a UC50 digital camera using cellSense software (Olympus, Center Valley, PA) at equal exposure times.

### In-cell Western blot

Subconfluent cells grown in 96-well opaque clear bottom cell culture plates were washed with PBS and fixed with 4% paraformaldehyde for 20 min. Cells were permeabilized with 0.1% Triton X-100 and endogenous peroxidase was quenched with H_2_O_2_ and NaN_3_ for 20 min. Cells were blocked with normal goat serum for 1 h and incubated with primary antibody at a 1:100 dilution overnight followed by washing as above and addition of secondary antibody at 1:1000 for 1 h. Blots were developed using West Femto (Pierce, Thermo Fisher Scientific), and chemiluminescence was read using a Synergy HT microplate reader with Gen5 software (BioTek, Winooski, VT) and imaged with ChemiDoc imaging system (Bio-Rad). Cells were then washed with PBS, stained with Janus Green stain for 1 min, washed and eluted in 100% ethanol. Absorbance was read at 594 nm. Chemiluminescence signal was normalized per cell number, and the negative control (secondary antibody only) signal was subtracted from an average of three wells per antibody. Expression was then reported relative to the β-actin signal.

### RNA isolation and cDNA synthesis

RNA was isolated using the RNeasy kit (Qiagen, Valencia, CA) for animal tissue analysis and sequencing samples, and with the Tissue/cell total RNA mini kit (EZ BioResearch, St. Louis, MO) for inhibitor studies. Snap-frozen kidney tissues were lysed with RNeasy lysis (RTL) buffer (Qiagen) supplemented with β-mercaptoethanol and homogenized using a motorized pellet pestle (Kontes, Vineland, NJ) followed by centrifugation in the Qiashredder (Qiagen). Cultured NRK-52E cells were trypsinized, pelleted, and lysed with RTL buffer (Qiagen) supplemented with β-mercaptoethanol and passed 5 times through a 20-gauge needle. On-column DNase digestion was performed for both tissues and cells. RNA concentration and quality was determined by spectrophotometry on a Nanodrop 2000c and confirmed by agarose gel electrophoresis. cDNA was generated using the iScript cDNA Synthesis Kit (Bio-Rad) for initial MMP and TIMP screening, and the High-Capacity cDNA Reverse Transcription kit (Applied Biosystems, Life Technologies) was used for later experiments.

### Real-time polymerase chain reaction

Initial MMP and TIMP screening was performed using the iCycler iQ real-time polymerase chain reaction (PCR) detection system (Version 3.1; Bio-Rad) and iQ SYBR® Green Supermix (Bio-Rad). Genes of interest were targeted using specific RT² Real-Time PCR primer sets (SuperArray; SABiosciences, Qiagen). Relative quantitation was performed using the ΔΔCt method in which the quantity of target gene mRNA in each experimental sample (young, aged-AL or aged-CR) relative to an internal standard (ß-actin mRNA) is normalized to an arbitrary reference sample (Universal Rat Reference RNA; Stratagene) (Akintola et al. 2008). In subsequent experiments, we used custom primer/probe Taqman® Assays (Applied Biosystems, Life Technologies) and the Sso Fast mix (Bio-Rad) with the CFX96 Touch real-time PCR system (Bio-Rad). Analysis was performed using the ΔΔCt method relative to Casc3 and ß-actin.

### Illumina sequencing

RNA from the normal rat kidney parent cell-line NRK-52E, as well as cells stably expressing wild-type MMP7, active mutant MMP7, and control vector, was submitted for high-throughput sequencing. A mRNA-focused, bar-coded library was generated using the TruSeq kit (Illumina, San Diego, CA) and analyzed using the HiSeq 2000 platform from Illumina at the DNA Core Facility at the University of Missouri. The sequencing reaction yielded ∼7.5 Gb of data, corresponding to around 30 million 50-base reads per sample across the whole transcriptome. The Informatics Research Core Facility at the University of Missouri aligned the reads against the rat genome (Rattus norvegicus RGSC3.4; Ensemble, Hinxton, UK) and analyzed them using Bowtie (Langmead and Salzberg 2012), TopHat and Cufflinks (Trapnell et al. 2012) software. Differential expression values defined as fragments per kilobase of transcript per million mapped reads with a false discovery–corrected *P*-value equal or lower than 0.05 were considered significant. The raw data from our Illumina high-throughput sequencing has been deposited in the Sequence Read Archive (SRA) with the National Center for Biotechnology Information (Bethesda, MD) under the project PRJNA213322, accession number SRP02851, experiment MMP7, accession number SRX327868, and will be made available upon publication of this manuscript.

### Inhibitors

The inhibitors used in this study were all purchased from Calbiochem (Darmstadt, Germany): GM6001 (MMPs), LY294002 (PI3K), UO126 (MEK [mitogen-activated protein kinase kinase]), 4-amino-5-(4-chlorophenyl)-7-(dimethylethyl)pyrazolo[3,4-d]pyrimidine (PP2) (src), SB203580 (4-(4-Fluorophenyl)-2-(4-methylsulfinylphenyl)-5-(4-pyridyl)1H-imidazole) and 2-(4-Chlorophenyl)-4-(4-fluorophenyl)-5-pyridin-4-yl-1,2-dihydropyrazol-3-one (p38), FR180204 (ERK1/2), Staurosporine (PKA/protein kinase C [PKC]), KT5720 (PKA), and Bisindolylmaleimide I (PKC). Cells were grown in 6- or 12-well plates in full medium as described in Cell culture above. Upon reaching 90% confluency, cells were washed once with serum-free DMEM/F12 and treated with indicated concentrations of inhibitors in serum-free medium.

### Statistics

For mRNA expression, in-cell Western, and enzymatic assay results, a two-tailed *t*-test assuming two-sample equal variance was performed with *P*-values <0.05 considered statistically significant.

## Results

### Age-related overexpression of MMP-7

**Figure 1:**
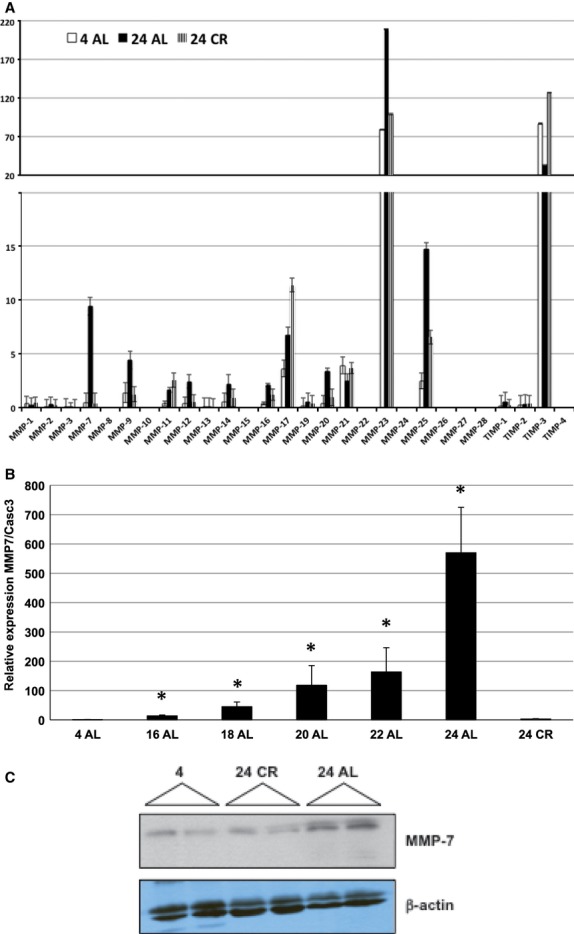
Age-dependent changes in MMP/TIMP expression in the kidney. (A) Relative expression of MMPs and TIMPs in young (4 AL), old (24 AL), and calorie-restricted animals (24 CR) as determined by real-time PCR. ß-actin was used as the reference gene. Expression of MMP-2, -3, -7, -9, -12, -13, -14, -16, -17, -19, -20, -23, and -25, as well as TIMP-1 changed significantly as a function of age. Of these, the increased expression of MMP-2, -7, -9, -12, -13, -14, -16, -20, -23, and -25 was attenuated by caloric restriction, as was TIMP-1, with *P* < 0.05. (B) MMP-7 expression in aging rat kidneys is significantly increased as early as 16 months. *P < 0.05. (C) MMP-7 protein expression is increased in the 24-month-old rat kidney, but not CR controls. Each lane represents a lysate from an individual animal.

Given the importance of MMPs in acute and chronic renal pathophysiologies (Catania et al. 2007), we examined the mRNA expression of all MMPs and TIMPs in young (4 month-old), aged, 24-month-old AL fed, and aged CR rat kidneys by quantitative PCR. Using rat-specific primers, we found expression of many MMPs that have not yet been linked to the kidney, including MMP-1, -16, -17, -20, -21, and -25 (Fig. 1A). In contrast to a previous report investigating human MMP-2 and MMP-24 (Romanic et al. 2001), expression of MMP-15 and -24 was not detected in the rat kidney. Importantly, we identified several MMPs whose gene expression was significantly changed as a function of aging, including MMP-2, -3, -7, -9, -12, -13, -14, -16, -17, -19, -20, -23, and -25, as well as TIMP-1. Of these, the increased expression of MMP-2, -7, -9, -12, -13, -14, -16, -20, -23, and -25 was attenuated by caloric restriction, as was TIMP-1. As MMP-7 exhibited the most dramatic increase in the aged animals and is overexpressed in the aging human kidney (Rodwell et al. 2004; Melk et al. 2005), we examined MMP-7 expression over an extensive time course. At 16 months expression was significantly upregulated, and increased to over 500-fold upregulation in 2-year-old animals (Fig. 1B). Importantly, increased gene expression correlated with increased protein expression as assessed by Western blot (Fig. 1C). The temporal pattern of MMP-7 overexpression, and the finding that it is not overexpressed in caloric restriction controls, suggests that MMP-7 may play a pathogenic role in the development of chronic renal dysfunction.

### MMP-7 overexpression: collagen expression

**Figure 2:**
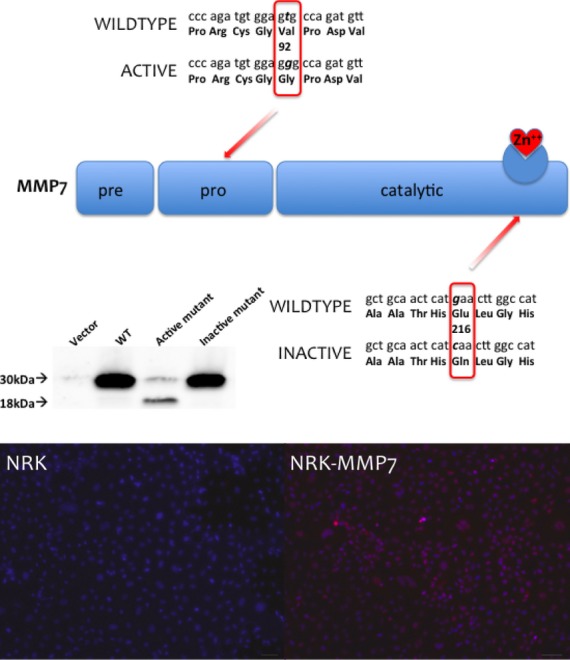
Generation of MMP-7 overexpressing cell lines. Normal rat kidney cells (NRK-52E) were stably transfected with full-length human MMP-7 (WT), a catalytically active mutant and an inactive mutant form. Immunofluorescence staining with anti-MMP-7 antibody in vector and MMP-7 WT overexpressing cells, DAPI counterstain (bottom panels). Concentrated conditioned medium immunoblotted with anti-MMP-7 antibody shows bands for proform ∼30 kDa and active form ∼18 kDa (insert).

In order to delineate the effects of MMP-7 overexpression in the kidney, we stably overexpressed MMP-7 in NRK-52E cells. As epithelial cells do not activate MMP-7 in vitro (Witty et al. 1994), we overexpressed wild-type MMP-7, an active mutant of MMP-7, and a catalytically inactive mutant. The active mutant has a point mutation resulting in a valine to glycine substitution at position 92 (Fig. 2). This mutation in the prodomain allows for an autocatalytic cleavage of the zymogen to produce a catalytically active MMP-7. The inactive mutant has a point mutation in the catalytic domain at position 216. Overexpressed MMP-7 was detectable in the NRK-52E cells and was secreted into the medium (Fig. 2). In conditioned medium from wild-type and the inactive mutant overexpressing NRK-52E cells, only the 30 kDa zymogen was visible on the Western blot. Expression of the active form was lower as determined by real-time PCR and Western blot, and bands representing both the 30 kDa pro- and a 18 kDa active form were detected. Each of the MMP-7 overexpressing cells exhibited comparable doubling times, which were shorter than those of the parent NRK-52E cell line, probably due to the strong cytomegalovirus promoter in the vector (data not shown). It is important to note that the relative expression of pro-MMP-7 appears to be higher in the wild-type and inactive mutant constructs than in the active mutant, which still expressed pro-MMP-7.

**Figure 3:**
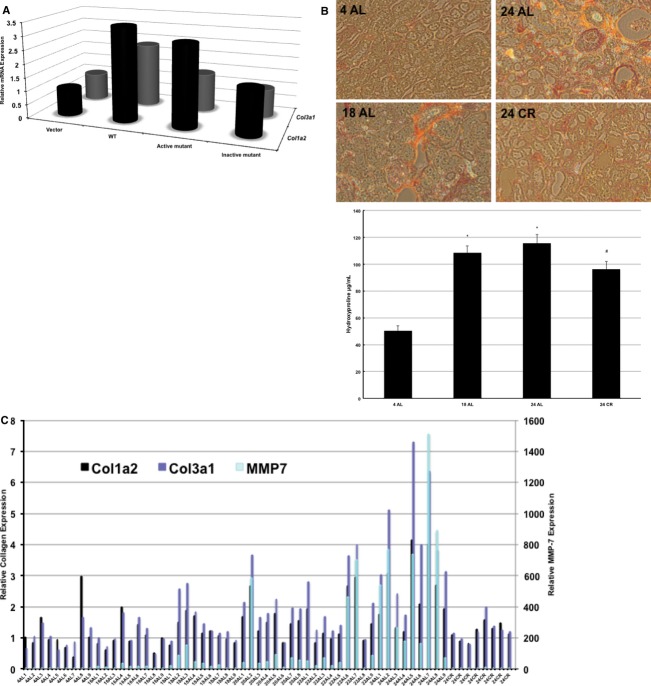
Relationship between MMP-7 and collagen expression. (A) *Col1a2* and *Col3a1* expression changes in MMP-7 overexpressing cell as determined by real-time PCR. Casc3 was used as the reference gene. The upregulation determined by Illumina sequencing was 3.9- and 2.1-fold for *Col1a2* and *Col3a1* in WT cells, and 5.0 and 1.4 in active mutant MMP-7 cells (A1) compared to vector control. (B) Fibrotic changes are visualized by sirius red staining of collagen deposition. Caloric-restricted (CR) 24-month-old rats are comparable to young, 4-month control animals (top panels). Confirmation of increased collagen levels in older animals as determined by the hydroxyproline assay (bottom graph). *P < 0.05 relative to 4 AL, #relative to 24 AL. (C) *Col1a2* and *Col3a1* expression (left *y*-axis) correlates with MMP-7 expression (right *y*-axis) in individual F344 rats and increases with age as determined by real-time PCR. Casc3 was used as the reference gene.

High-throughput sequencing of mRNA libraries generated from MMP-7 overexpressing cells yielded promising target genes, including *Col1a2* and *Col3a1*, interestingly, in both the WT and active mutant MMP-7 overexpressing cells (Fig. 3A; Table1). While WT overexpressing cells had the largest increase in collagen expression, the catalytic activity of MMP-7 may be important given the findings that the active mutant cells also were characterized by collagen overexpression and that this effect was significantly decreased in the inactive mutant cells. Increased collagen deposition is characteristic of the aging rat kidney (Fig. 3B). As expected, expression of both collagens increased with age and paralleled the temporal changes in MMP-7 overexpression (Fig. 3C).

**Table 1:** Vector vs MMP-7 WT

Test_id	Gene_id	Gene	Locus	Vector	WT	Log2 (fold_change)	Test_stat	*P*-value	*q*-value	Fold change	
ENSRNOT00000046954	ENSRNOG00000034295	–	6:6947525–6995752	12.881	1.0758	−3.58177	5.0234	5.08E−07	0.000179958	0.08	no protein, pseudogene in Renal function QTL16
ENSRNOT00000017623	ENSRNOG00000012939	ABCA7_RAT	7:11203982–11222960	1.20463	3.87364	1.6851	−3.89959	9.64E−05	0.0143744	3.22	ATP-binding cassette sub-family A member 7
ENSRNOT00000064886	ENSRNOG00000012939	ABCA7_RAT	7:11203982–11222960	2.86713	0.324656	−3.14262	3.83916	0.000123456	0.0175413	0.11	ABC transporter, conserved site, ATPase, AAA+type, core, ABC transporter-like
ENSRNOT00000064572	ENSRNOG00000001404	Agfg2	12:19716014–19752171	0	3.67817	1.79769e+308	1.79769e+308	1.74E−05	0.0036515	up	Arf-GAP domain and FG repeats-containing protein 2
ENSRNOT00000025258	ENSRNOG00000018598	Ankrd1	1:240316122–240324804	40.6488	10.8954	−1.89949	7.22162	5.14E−13	7.21E−10	0.27	ankyrin repeat domain 1 (cardiac muscle)
ENSRNOT00000049698	ENSRNOG00000006094	Cd44	3:88022982–88110352	11.8848	1.42914	−3.0559	3.65923	0.000252976	0.0303702	0.12	CD44
ENSRNOT00000036025	ENSRNOG00000021285	CELSR1	7:123900402–124036122	7.81533	13.788	0.819038	−3.73805	0.00018545	0.0239456	1.76	cadherin, EGF LAG seven-pass G-type receptor 1 (flamingo homolog, Drosophila)
ENSRNOT00000016423	ENSRNOG00000011292	Col1a2	4:29393550–29428568	2.90803	11.3761	1.96789	−6.34732	2.19E−10	1.75E−07	3.91	Collagen alpha-2(I) chain
ENSRNOT00000004956	ENSRNOG00000003357	Col3a1	9:44281581–44317827	115.743	243.059	1.07038	−4.5815	4.62E−06	0.00121382	2.10	collagen, type III, alpha 1
ENSRNOT00000019501	ENSRNOG00000014350	Cyr61	2:243824302–243827262	331.587	183.356	−0.854743	3.97211	7.12E−05	0.0113395	0.55	Cysteine-rich angiogenic inducer 61
ENSRNOT00000057522	ENSRNOG00000030213	D3ZEY5_RAT	8:72196263–72365798	0.674155	0	−1.79769e+308	1.79769e+308	0.00028722	0.0334497	not expressed	SF-assemblin, Vacuolar protein sorting-associated protein
ENSRNOT00000047772	ENSRNOG00000037380	D3ZQW7_RAT	1:88001743–88067218	101.201	7.59185	−3.73663	9.86554	0	0	0.08	Ribosomal protein S5
ENSRNOT00000044096	ENSRNOG00000006028	D4A709_RAT	7:127403424–127423259	3.1215	7.69981	1.30258	−3.63814	0.00027461	0.0324075	2.47	Tubulin gamma complex associated protein 6, Tubgcp6
ENSRNOT00000051316	ENSRNOG00000012209	E9PTG4_RAT	15:38658775–38687199	4.36822	0	−1.79769e+308	1.79769e+308	6.25E−05	0.0101695	not expressed	Cytidine deaminase-like, APOBEC/CMP deaminase, zinc-binding, CMP/dCMP deaminase, zinc-binding
ENSRNOT00000044776	ENSRNOG00000018121	E9PTW0_RAT	2:58667033–58720040	0.746746	85.5269	6.83962	−11.8052	0	0	114.53	Ribosomal protein S5, N-terminal
ENSRNOT00000019361	ENSRNOG00000014361	Edn1	17:28303885–28309775	54.8802	23.6102	−1.21687	4.88724	1.02E−06	0.000329694	0.43	endothelin 1
ENSRNOT00000013608	ENSRNOG00000009439	Eef1a1	8:83463586–83466816	3348.41	3292.51	−0.0242881	4.72225	2.33E−06	0.000676657	0.98	eukaryotic translation elongation factor 1 alpha 1
ENSRNOT00000032780	ENSRNOG00000001469	Eln	12:23033656–23076086	137.942	329.623	1.25675	−4.73083	2.24E−06	0.000654139	2.39	elastin
ENSRNOT00000023825	ENSRNOG00000017719	F1M599_RAT	4:123811374–123820389	0.137075	13.1672	6.58584	−7.86897	3.55E−15	6.95E−12	96.06	novel protein, similar to glutamate receptor, ionotropic, N-methyl D-aspartate-like 1A (Grinl1a)
ENSRNOT00000052149	ENSRNOG00000019579	F1M6R5_RAT	8:61472271–61516975	3.44162	0	−1.79769e+308	1.79769e+308	0.000221007	0.0273718	not expressed	YjeF-related protein, N-terminal
ENSRNOT00000005709	ENSRNOG00000004290	Grb10	14:92814796–92911442	0	3.04998	1.79769e+308	1.79769e+308	0.000286745	0.0334497	up	Growth factor receptor-bound protein 10
ENSRNOT00000064187	ENSRNOG00000007000	Grhl2	7:72742858–72872350	0.0605663	0.653535	3.43168	−4.74934	2.04E−06	0.000605972	10.79	CP2 transcription factor, grainyhead-like 2 (Drosophila)
ENSRNOT00000015894	ENSRNOG00000011847	Grk4	14:81648002–81722480	0	1.66054	1.79769e+308	1.79769e+308	0.000179469	0.023406	uo	G protein-coupled receptor kinase 4
ENSRNOT00000016174	ENSRNOG00000012119	LOC690209	8:14245341–14246673	20.9934	6.87195	−1.61114	4.89442	9.86E−07	0.000319748	0.33	similar to NIMA (never in mitosis gene a) -related exp NPR3
ENSRNOT00000004684	ENSRNOG00000003532	Magea11	X:144114831–144120816	1.28968	22.0279	4.09425	−9.23241	0	0	17.08	Melanoma-associated antigen 11
ENSRNOT00000000169	ENSRNOG00000000156	Megf6	5:170848978–171078739	22.4684	40.0213	0.832874	−3.78871	0.000151431	0.020509	1.78	multiple EGF-like-domains 6
ENSRNOT00000067408	ENSRNOG00000006699	Mlh3	6:109280909–109318893	0	0.941301	1.79769e+308	1.79769e+308	0.000154966	0.0208462	up	DNA mismatch repair protein Mlh3
ENSRNOT00000046803	ENSRNOG00000007948	Nf2	14:85415141–85508807	0	4.22702	1.79769e+308	1.79769e+308	6.89E−05	0.0110418	up	neurofibromin 2 (merlin)
ENSRNOT00000046152	ENSRNOG00000021996	Nlrp4	1:66797942–66825101	0.336655	2.11805	2.6534	−4.92581	8.40E−07	0.000277953	6.29	NACHT, LRR and PYD domains-containing protein 4
ENSRNOT00000010779	ENSRNOG00000008141	Nppb	5:165062347–165063650	16.0836	3.25884	−2.30316	4.49445	6.98E−06	0.00172925	0.20	natriuretic peptide B
ENSRNOT00000060426	ENSRNOG00000010477	Pomt1	3:11348785–11366632	0	5.56385	1.79769e+308	1.79769e+308	0.000173499	0.0227703	up	Protein O-mannosyl-transferase 1
ENSRNOT00000055032	ENSRNOG00000013267	Pric285	3:170368820–170382086	0	0.57973	1.79769e+308	1.79769e+308	0.000232451	0.0285096	up	Peroxisomal proliferator-activated receptor A interacting complex 285
ENSRNOT00000052290	ENSRNOG00000032703	Rasgrp3	6:19808452–19871923	8.30593	4.20947	−0.980502	3.51371	0.000441899	0.0463619	0.51	Ras guanyl-releasing protein 3
ENSRNOT00000059819	ENSRNOG00000002144	Sec3l1	14:33883343–33920857	8.24032	0.596241	−3.78873	3.77394	0.00016069	0.0215016	0.07	exocyst complex component 1
ENSRNOT00000001916	ENSRNOG00000001414	Serpine1	12:20931995–20942374	62.8455	33.8617	−0.892153	4.18393	2.87E−05	0.00547625	0.54	Serpine 1
ENSRNOT00000063959	ENSRNOG00000020138	Slc4a3	9:74823768–74835860	2.77433	0	−1.79769e+308	1.79769e+308	1.44E−05	0.00314476	not expressed	Anion exchange protein 3
ENSRNOT00000039221	ENSRNOG00000026607	Tnfsf18	13:77136963–77145251	35.8281	8.85273	−2.0169	4.69464	2.67E−06	0.000754331	0.25	Tumor necrosis factor ligand superfamily member 18
ENSRNOT00000011530	ENSRNOG00000008717	–	6:127258746–127462319	68.0545	0.142029	−8.90436	10.8992	0	0	479.16	novel transcript within Urinary albumin excretion QTL 7
ENSRNOT00000033844	ENSRNOG00000021292	–	17:59022844–59275923	27.9701	6.97332	−2.00397	5.82617	5.67E−09	3.33E−06	4.01	retinoblastoma binding protein 4; similar to Chromatin assembly factor 1 subunit CG4236-PA
ENSRNOT00000034355	ENSRNOG00000026168	–	8:125535679–125536042	25.0815	0	−1.79769e+308	−1.79769e+308	1.70E−05	0.00357667	up	Novel retrotransposed, within Collagen induced arthritis QTL 6
ENSRNOT00000017623	ENSRNOG00000012939	ABCA7_RAT	7:11203982–11222960	3.5788	1.20463	−1.57089	3.58895	0.000332008	0.0372815	2.97	ATP-binding cassette sub-family A member 7
ENSRNOT00000064572	ENSRNOG00000001404	Agfg2	12:19716014–19752171	2.82724	0	−1.79769e+308	−1.79769e+308	0.000109477	0.0159041	up	arf-GAP domain and FG repeats-containing protein 2
ENSRNOT00000025258	ENSRNOG00000018598	Ankrd1	1:240316122–240324804	19.6078	40.6488	1.05179	−4.35548	1.33E−05	0.00294741	0.48	Ankyrin repeat domain-containing protein 1
ENSRNOT00000026058	ENSRNOG00000019253	Bcar1	19:41646189–41669234	24.8368	53.8451	1.11634	−3.54057	0.00039926	0.0429581	0.46	Breast cancer anti-estrogen resistance protein 1
ENSRNOT00000049698	ENSRNOG00000006094	Cd44	3:88022982–88110352	2.12927	11.8848	2.48069	−3.68657	0.000227297	0.0280383	0.18	CD44 antigen
ENSRNOT00000016423	ENSRNOG00000011292	Col1a2	4:29393550–29428568	14.5862	2.90803	−2.32649	7.61096	2.73E−14	4.69E−11	5.02	Collagen alpha-2(I) chain
ENSRNOT00000068558	ENSRNOG00000033169	Cpeb4	10:15968781–16026700	2.62906	0	−1.79769e+308	−1.79769e+308	5.69E−05	0.00947819	up	cytoplasmic polyadenylation element-binding protein 4
ENSRNOT00000029132	ENSRNOG00000030213	D3ZEY5_RAT	8:72196263–72365798	1.86717	0.48052	−1.95819	3.49363	0.000476492	0.0491335	3.89	similar to SF-assemblin, Vacuolar protein sorting-associated protein
ENSRNOT00000057522	ENSRNOG00000030213	D3ZEY5_RAT	8:72196263–72365798	0	0.674155	1.79769e+308	1.79769e+308	0.00028722	0.0334497	not expressed	similar to vsp13c, SF-assemblin, Vacuolar protein sorting-associated protein
ENSRNOT00000067052	ENSRNOG00000027569	D3ZJK6_RAT	7:110316544–110793515	1.4168	9.28808	2.71274	−3.55196	0.000382373	0.0415763	0.15	trafficking protein particle complex 9
ENSRNOT00000047364	ENSRNOG00000000922	D3ZTR4_RAT	12:28003490–28028905	48.1732	22.6988	−1.08561	3.77329	0.000161109	0.0215162	2.12	similar to SUMF2 sulfatase modifying factor 2
ENSRNOT00000013608	ENSRNOG00000009439	Eef1a1	8:83463586–83466816	3263.17	3348.41	0.0371986	−7.19883	6.07E−13	8.36E−10	0.97	Elongation factor 1-alpha 1
ENSRNOT00000023825	ENSRNOG00000017719	F1M599_RAT	4:123811374–123820389	12.497	0.137075	−6.51047	7.7679	7.99E−15	1.47E−11	91.17	similar to polymerase (RNA) II (DNA directed) polypeptide M
ENSRNOT00000056983	ENSRNOG00000006738	Fbxo32	7:94909567–94942444	0	1.83838	1.79769e+308	1.79769e+308	0.000205778	0.02596	not expressed	F-box only protein 32
ENSRNOT00000018788	ENSRNOG00000014029	Klhl13	X:10344240–10424664	13.0508	4.89107	−1.41592	3.49066	0.000481835	0.0494691	2.67	kelch-like 13,BTB and kelch domain containing 2
ENSRNOT00000063868	ENSRNOG00000014029	Klhl13	X:10344240–10424664	0	3.17109	1.79769e+308	1.79769e+308	0.000349141	0.0387204	not expressed	kelch-like 13
ENSRNOT00000007696	ENSRNOG00000005869	LOC498453	15:11865466–12045333	8.80749	0	−1.79769e+308	−1.79769e+308	4.27E−07	0.000156012	up	similar to transcription elongation factor A 1 isoform 2
ENSRNOT00000016991	ENSRNOG00000012495	Podxl	4:58611905–58658598	0.458957	0.0620132	−2.88771	4.09568	4.21E−05	0.00742765	7.40	Podocalyxin
ENSRNOT00000000725	ENSRNOG00000000593	Rev3l	20:43870508–44042379	3.12343	0.529002	−2.56179	4.99287	5.95E−07	0.000206406	5.90	DNA polymerase zeta catalytic subunit, REV3-like
ENSRNOT00000063936	ENSRNOG00000033389	Susd2	20:13435256–13442683	2.35239	0	−1.79769e+308	−1.79769e+308	7.25E−07	0.000244074	up	sushi domain-containing protein 2
ENSRNOT00000046954	ENSRNOG00000034295	–	6:6947525–6995752	11.0197	1.0758	−3.35661	4.66138	3.14E−06	0.000872923	10.24	novel transcript, within intron of Potassium voltage-gated channel subfamily G member 3, Kcng3
ENSRNOT00000034355	ENSRNOG00000026168	–	8:125535679–125536042	25.0815	0.409245	−5.93751	5.19284	2.07E−07	8.40E−05	61.29	novel transcript, retrotransposed, no protein prouct
ENSRNOT00000033844	ENSRNOG00000021292	–	17:59022844–59275923	27.9701	8.30695	−1.75149	5.35403	8.60E−08	3.87E−05	3.37	retinoblastoma binding protein 4; similar to Chromatin assembly factor 1 subunit CG4236-PA
ENSRNOT00000011530	ENSRNOG00000008717	–	6:127258746–127462319	68.0545	0.146856	−8.85614	11.2454	0	0	463.41	novel transcript within Urinary albumin excretion QTL 7
ENSRNOT00000068558	ENSRNOG00000033169	Cpeb4	10:15968781–16026700	2.62906	0	−1.79769e+308	−1.79769e+308	5.69E−05	0.00947819	up	cytoplasmic polyadenylation element-binding protein 4
ENSRNOT00000018888	ENSRNOG00000014048	CYLD_RAT	19:19617011–19644586	0	4.1787	1.79769e+308	1.79769e+308	1.65E−05	0.00350119	not expressed	Ubiquitin carboxyl-terminal hydrolase CYLD
ENSRNOT00000012501	ENSRNOG00000030213	D3ZEY5_RAT	8:72196263–72365798	1.60088	3.96778	1.30946	−4.14081	3.46E−05	0.00634453	0.40	similar to VPS13C, vacuolar protein sorting 13 homolog C (S. cerevisiae)
ENSRNOT00000067052	ENSRNOG00000027569	D3ZJK6_RAT	7:110316544–110793515	1.4168	9.74414	2.7819	−3.62734	0.000286351	0.0334497	0.15	trafficking protein particle complex 9
ENSRNOT00000047772	ENSRNOG00000037380	D3ZQW7_RAT	1:88001743–88067218	104.886	7.59185	−3.78823	10.0102	0	0	13.82	Uncharacterized protein, similar to ribosomal protein S5
ENSRNOT00000042105	ENSRNOG00000032471	D3ZYV8_RAT	14:112127247–112174996	1.08329	7.32505	2.75741	−3.49259	0.000478367	0.0492765	0.15	ankyrin repeat and SOCS box protein 3
ENSRNOT00000044096	ENSRNOG00000006028	D4A709_RAT	7:127403424–127423259	3.01893	7.69981	1.35079	−3.71936	0.000199731	0.0253235	0.39	tubulin, gamma complex associated protein 6
ENSRNOT00000065458	ENSRNOG00000002152	Dcun1d4	14:37051132–37128945	1.84175	6.03693	1.71274	−3.94183	8.09E−05	0.0125547	0.31	DCN1-like protein 4; defective in cullin neddylation 1, domain containing 4
ENSRNOT00000051316	ENSRNOG00000012209	E9PTG4_RAT	15:38658775–38687199	5.20533	0	−1.79769e+308	−1.79769e+308	1.18E−05	0.00268048	up	cytidine and dCMP deaminase domain containing 1
ENSRNOT00000044776	ENSRNOG00000018121	E9PTW0_RAT	2:58667033–58720040	0.305094	85.5269	8.13098	−10.5805	0	0	0.00	Ribosomal protein S5
ENSRNOT00000020573	ENSRNOG00000015133	F1M0L3_RAT	8:47759174–47834586	2.13606	5.07054	1.24719	−4.08208	4.46E−05	0.00781665	0.42	Myeloid/lymphoid or mixed-lineage leukemia (Mapped)Uncharacterized protein
ENSRNOT00000064187	ENSRNOG00000007000	Grhl2	7:72742858–72872350	0.106551	0.653535	2.61672	−3.99644	6.43E−05	0.0104084	0.16	grainyhead-like protein 2 homolog
ENSRNOT00000004460	ENSRNOG00000003345	LOC302762	X:77009878–77012222	0.088109	0.749615	3.08879	−3.83252	0.000126838	0.017912	0.12	PREDICTED: DDB1- and CUL4-associated factor 8-like, similar to plasmacytoma expressed transript 2
ENSRNOT00000007696	ENSRNOG00000005869	LOC498453	15:11865466–12045333	8.80749	0	−1.79769e+308	−1.79769e+308	4.27E−07	0.000156012	up	similar to transcription elongation factor A 1 isoform 2; transcription elongation factor A (SII) 1
ENSRNOT00000016174	ENSRNOG00000012119	LOC690209	8:14245341–14246673	20.3598	6.87195	−1.56693	4.74563	2.08E−06	0.000614553	2.96	similar to NIMA (never in mitosis gene a) -related expressed kinase 2
ENSRNOT00000004684	ENSRNOG00000003532	Magea11	X:144114831–144120816	2.56517	22.0279	3.1022	−7.82805	4.88E−15	9.30E−12	0.12	melanoma-associated antigen 11, similar to mage-k1
ENSRNOT00000060426	ENSRNOG00000010477	Pomt1	3:11348785–11366632	0	5.56385	1.79769e+308	1.79769e+308	0.000173499	0.0227703	not expressed	Protein O-mannosyl-transferase 1
ENSRNOT00000049814	ENSRNOG00000004819	Porcn	X:26317406–26330171	0	3.49927	1.79769e+308	1.79769e+308	0.000282208	0.0330933	not expressed	porcupine homolog
ENSRNOT00000055971	ENSRNOG00000021780	Rad51l3	10:71092821–71107418	1.99246	0	−1.79769e+308	−1.79769e+308	0.000300691	0.0346468	up	DNA repair protein RAD51 homolog 4
ENSRNOT00000066106	ENSRNOG00000008340	RGD1309779	8:67558903–67563530	0	8.1175	1.79769e+308	1.79769e+308	0.000454238	0.0474384	not expressed	Antifreeze protein, type I
ENSRNOT00000063936	ENSRNOG00000033389	Susd2	20:13435256–13442683	2.35239	0	−1.79769e+308	−1.79769e+308	7.25E−07	0.000244074	up	sushi domain-containing protein 2
ENSRNOT00000009762	ENSRNOG00000007428	Ypel4	3:67945701–67947571	0	2.48631	1.79769e+308	1.79769e+308	9.47E−05	0.0141986	not expressed	Protein yippee-like 4
ENSRNOT00000048322	ENSRNOG00000029947	–	18:24386615–24444446	0.247115	285.439	10.1738	−15.0338	0	0	1155.085689	
ENSRNOT00000059785	ENSRNOG00000027022	–	19:32261770–32521913	4.3896	40.097	3.19133	−3.74977	0.000176997	0.0231596	9.134545289	
ENSRNOT00000041892	ENSRNOG00000031706	–	8:24852588–24853338	84.6196	39.8219	−1.08743	4.25664	2.08E−05	0.00423136	0.47059901	
ENSRNOT00000048837	ENSRNOG00000033307	–	17:33352145–33352895	175.978	78.2828	−1.16863	5.17046	2.34E−07	9.39E−05	0.444844242	
ENSRNOT00000016040	ENSRNOG00000011964	Abcd4	6:108660718–108681707	6.94143	14.753	1.08771	−3.61808	0.000296801	0.0343124	2.125354574	
ENSRNOT00000024084	ENSRNOG00000017786	Acta1	19:54081497–54084508	2.69603	9.55081	1.82479	−4.1097	3.96E−05	0.00709345	3.542545892	
ENSRNOT00000013286	ENSRNOG00000009951	Aif1l	3:11053195–11078079	31.4084	68.946	1.13432	−5.28738	1.24E−07	5.35E−05	2.195145248	
ENSRNOT00000029137	ENSRNOG00000010877	Alg9	8:54131721–54194200	5.6306	0	−1.79769e+308	−1.79769e+308	2.91E−06	0.000811706	0	
ENSRNOT00000022585	ENSRNOG00000016678	Angptl2	3:12147164–12345170	34.3392	15.3835	−1.15848	4.48941	7.14E−06	0.00175758	0.447986558	
ENSRNOT00000025258	ENSRNOG00000018598	Ankrd1	1:240316122–240324804	8.98593	40.6488	2.17747	−7.96134	1.78E−15	3.66E−12	4.523605236	
ENSRNOT00000065912	ENSRNOG00000007110	Ankrd6	5:49098943–49238039	1.20956	3.2422	1.42249	−3.50276	0.000460467	0.0478941	2.680478852	
ENSRNOT00000027464	ENSRNOG00000020270	Anxa8	16:9715643–9730577	12.7852	25.2696	0.982934	−3.70747	0.000209344	0.0262431	1.976472797	
ENSRNOT00000002857	ENSRNOG00000002095	Arhgap24	14:8026135–8346326	0.169448	2.00403	3.56399	−5.67541	1.38E−08	7.60E−06	11.82681413	
ENSRNOT00000021801	ENSRNOG00000016066	Bambi	17:62654079–62658885	32.1927	58.4287	0.859944	−3.58421	0.000338099	0.0377975	1.814967368	
ENSRNOT00000014267	ENSRNOG00000010698	Car1	2:88198729–88210693	1.61929	7.02468	2.11707	−4.11301	3.91E−05	0.0070206	4.338123499	
ENSRNOT00000014180	ENSRNOG00000010079	Car3	2:88105881–88114721	9.18183	20.6693	1.17064	−3.76407	0.00016717	0.0221871	2.251108984	
ENSRNOT00000008722	ENSRNOG00000006411	Cav2	4:42932126–42939501	20.3781	46.4948	1.19005	−5.04677	4.49E−07	0.000162733	2.281606234	
ENSRNOT00000027084	ENSRNOG00000019939	CCND2_RAT	4:163524290–163546640	82.9549	153.687	0.889592	−4.0537	5.04E−05	0.00861661	1.852657287	
ENSRNOT00000023977	ENSRNOG00000017819	Cd14	18:29374596–29376328	69.2244	34.7166	−0.995653	4.34566	1.39E−05	0.00304593	0.501508139	
ENSRNOT00000021268	ENSRNOG00000015821	Cd2	2:196332589–196346221	1.12977	4.9592	2.13408	−3.51066	0.00044699	0.0468076	4.389566018	
ENSRNOT00000038016	ENSRNOG00000027456	Cdc42bpg	1:209083956–209103885	2.00095	5.28006	1.39987	−4.52326	6.09E−06	0.00154558	2.638776581	
ENSRNOT00000000628	ENSRNOG00000000521	Cdkn1a	20:7379385–7385595	229.484	392.911	0.77581	−3.70105	0.000214713	0.0268193	1.712149867	
ENSRNOT00000035930	ENSRNOG00000026604	Cercam	3:8857698-−-8871398	0	0.866262	1.79769e+308	1.79769e+308	0.000195831	0.0249543	#DIV/0!	
ENSRNOT00000028440	ENSRNOG00000020952	Cgn	2:189644802–189663203	8.43665	18.6037	1.14085	−4.70097	2.59E−06	0.000736554	2.205105107	
ENSRNOT00000048519	ENSRNOG00000000463	Col11a2	20:4924451–4953310	0.459021	0	−1.79769e+308	−1.79769e+308	5.68E−05	0.0094764	0	
ENSRNOT00000016423	ENSRNOG00000011292	Col1a2	4:29393550–29428568	15.5644	2.90803	−2.42013	7.94696	2.00E−15	4.08E−12	0.186838555	
ENSRNOT00000009985	ENSRNOG00000007234	CP51A_RAT	4:26752355–26770318	47.6671	24.0287	−0.988237	4.40456	1.06E−05	0.00244674	0.504094019	
ENSRNOT00000068389	ENSRNOG00000016752	Crispld2	19:50283063–50378028	0.540175	0	−1.79769e+308	−1.79769e+308	0.000151645	0.020509	0	
ENSRNOT00000025222	ENSRNOG00000018659	Csf1	2:203292764–203307965	21.2806	38.8832	0.869608	−4.00455	6.21E−05	0.0101341	1.827166527	
ENSRNOT00000017310	ENSRNOG00000012896	Cyp2c11	1:243281319–243320945	0.956294	3.95536	2.04828	−3.9791	6.92E−05	0.0110682	4.136133867	
ENSRNOT00000057522	ENSRNOG00000030213	D3ZEY5_RAT	8:72196263–72365798	0	0.674155	1.79769e+308	1.79769e+308	0.00028722	0.0334497	#DIV/0!	
ENSRNOT00000045362	ENSRNOG00000028910	D3ZKN0_RAT	3:105260698–105266080	15.4701	31.0881	1.00688	−3.9696	7.20E−05	0.0114333	2.009560378	
ENSRNOT00000047772	ENSRNOG00000037380	D3ZQW7_RAT	1:88001743–88067218	8.48807	101.201	3.57565	−9.66754	0	0	11.92273391	
ENSRNOT00000067423	ENSRNOG00000019770	D4A0X9_RAT	1:138189344–138202803	11.3313	22.6131	0.996847	−3.53674	0.000405097	0.0434354	1.995631569	
ENSRNOT00000022899	ENSRNOG00000031743	D4A6I2_RAT	2:240527532–240541078	2.47696	0	−1.79769e+308	−1.79769e+308	3.74E−05	0.00678173	0	
ENSRNOT00000007750	ENSRNOG00000005887	D4A6I7_RAT	7:112829665–112831373	19.5637	67.7496	1.79203	−4.05538	5.01E−05	0.00856585	3.46302591	
ENSRNOT00000044096	ENSRNOG00000006028	D4A709_RAT	7:127403424–127423259	7.54706	3.1215	−1.27368	3.6294	0.000284081	0.0332428	0.413604768	
ENSRNOT00000019301	ENSRNOG00000014293	D4AAV5_RAT	19:19757067–19833022	4.45908	0.611558	−2.86619	6.42977	1.28E−10	1.09E−07	0.137148919	
ENSRNOT00000035977	ENSRNOG00000025883	D4AEE6_RAT	20:5379965–5391529	0.622497	2.91801	2.22885	−3.51577	0.000438474	0.0460199	4.687588856	
ENSRNOT00000009402	ENSRNOG00000006787	Dhcr24	5:127637375–127662621	61.0847	26.911	−1.18261	4.89884	9.64E−07	0.000313358	0.440552217	
ENSRNOT00000012532	ENSRNOG00000009291	Dnase1l3	15:18909362–18935342	2.46494	0.376542	−2.71067	4.16631	3.10E−05	0.00581839	0.152759094	
ENSRNOT00000044776	ENSRNOG00000018121	E9PTW0_RAT	2:58667033–58720040	95.2285	0.746746	−6.99463	12.0933	0	0	0.007841623	
ENSRNOT00000013608	ENSRNOG00000009439	Eef1a1	8:83463586–83466816	3935.43	3348.41	−0.233048	47.2063	0	0	0.850837139	
ENSRNOT00000026303	ENSRNOG00000019422	Egr1	18:27343566–27347352	7.64836	1.50755	−2.34294	5.79267	6.93E−09	3.97E−06	0.197107615	
ENSRNOT00000032780	ENSRNOG00000001469	Eln	12:23033656–23076086	314.631	137.942	−1.18959	4.48949	7.14E−06	0.00175758	0.438424694	
ENSRNOT00000003615	ENSRNOG00000002664	Emp2	10:5311156–5348037	21.5151	44.9042	1.0615	−4.72352	2.32E−06	0.000673162	2.087101617	
ENSRNOT00000005612	ENSRNOG00000004078	Eno3	10:57536964–57542311	32.2663	74.9383	1.21568	−5.23498	1.65E−07	6.89E−05	2.322494367	
ENSRNOT00000019519	ENSRNOG00000013994	Enpp1	1:21223677–21287411	72.8494	33.2307	−1.1324	5.25403	1.49E−07	6.29E−05	0.456156125	
ENSRNOT00000025663	ENSRNOG00000018982	Entpd3	8:125542933–125573945	0.250548	1.19274	2.25112	−3.66302	0.000249263	0.0299892	4.760524929	
ENSRNOT00000019720	ENSRNOG00000014367	Ephb6	4:69316599–69331856	1.05752	0.0547861	−4.27073	3.85539	0.000115546	0.0166214	0.051806207	
ENSRNOT00000000737	ENSRNOG00000000599	F1LTF8_RAT	20:43180812–43260729	0.0531117	0.35923	2.7578	−3.771	0.000162597	0.0216734	6.763669775	
ENSRNOT00000040881	ENSRNOG00000015133	F1M0L3_RAT	8:47759174–47834586	2.94978	1.10333	−1.41874	3.57262	0.000353423	0.0390706	0.374038064	
ENSRNOT00000059887	ENSRNOG00000039146	F1M2U4_RAT	11:53424952–53653313	11.4671	0.699976	−4.03405	4.7502	2.03E−06	0.00060442	0.061042112	
ENSRNOT00000002814	ENSRNOG00000002053	F1M3H3_RAT	14:14309716–14565184	2.77457	5.27226	0.926158	−3.78463	0.00015394	0.0207583	1.90020796	
ENSRNOT00000007876	ENSRNOG00000005986	F1M5X9_RAT	4:37617356–37880157	0.456827	6.47324	3.82477	−5.07286	3.92E−07	0.000146385	14.17000309	
ENSRNOT00000052149	ENSRNOG00000019579	F1M6R5_RAT	8:61472271–61516975	0	3.44162	1.79769e+308	1.79769e+308	0.000221007	0.0273718	#DIV/0!	
ENSRNOT00000003320	ENSRNOG00000002403	Fam129a	13:66467072–66620137	4.96483	22.8277	2.20097	−8.30368	0	0	4.597881498	
ENSRNOT00000056983	ENSRNOG00000006738	Fbxo32	7:94909567–94942444	0	1.83838	1.79769e+308	1.79769e+308	0.000205778	0.02596	#DIV/0!	
ENSRNOT00000004183	ENSRNOG00000003136	Fcrla	13:86775184–86785281	1.44194	7.54945	2.38836	−4.66781	3.04E−06	0.000848609	5.235620067	
ENSRNOT00000065065	ENSRNOG00000043377	Fdps	2:181168902–181177792	172.389	95.1902	−0.856779	3.89786	9.70E−05	0.014446	0.552182564	
ENSRNOT00000029284	ENSRNOG00000016050	Fgfr1	16:70869973–70910045	5.33782	1.3706	−1.96145	3.61352	0.000302063	0.0347329	0.256771491	
ENSRNOT00000023144	ENSRNOG00000016818	Fgfr3	14:82683190–82697229	17.3903	61.7206	1.82747	−4.63187	3.62E−06	0.000987366	3.549139463	
ENSRNOT00000006454	ENSRNOG00000004874	Flrt3	3:128922732–128934866	13.5093	37.2757	1.46429	−6.1661	7.00E−10	4.96E−07	2.759262138	
ENSRNOT00000004382	ENSRNOG00000003183	Fmod	13:46987713–46998330	2.95122	0.329223	−3.16417	5.81051	6.23E−09	3.61E−06	0.111554882	
ENSRNOT00000010712	ENSRNOG00000008015	Fos	6:109559134–109562001	43.9474	14.1807	−1.63185	6.18678	6.14E−10	4.48E−07	0.322674379	
ENSRNOT00000045765	ENSRNOG00000018500	Frmd4a	17:84783243–85068101	3.93629	1.40724	−1.48396	3.55715	0.000374898	0.0409076	0.357504147	
ENSRNOT00000004725	ENSRNOG00000003512	Gabra1	10:27258816–27313725	29.5779	12.5073	−1.24175	5.32286	1.02E−07	4.53E−05	0.422859635	
ENSRNOT00000018252	ENSRNOG00000013090	Gadd45g	17:19230895–19232641	56.8067	113.132	0.993874	−4.31728	1.58E−05	0.00338489	1.991525648	
ENSRNOT00000047019	ENSRNOG00000004290	Grb10	14:92814796–92911442	30.2417	15.0891	−1.00304	4.47503	7.64E−06	0.00186894	0.498950125	
ENSRNOT00000023554	ENSRNOG00000016552	Hmgcs1	2:51737089–51753895	46.0965	21.5714	−1.09554	4.82166	1.42E−06	0.000438176	0.467961776	
ENSRNOT00000028066	ENSRNOG00000020679	Icam1	8:20040164–20051949	56.3622	100.403	0.833005	−3.97193	7.13E−05	0.0113414	1.781388945	
ENSRNOT00000020144	ENSRNOG00000014835	Il1rl1	9:39577878–39624781	0.470228	5.58205	3.56936	−6.50577	7.73E−11	6.89E−08	11.87094346	
ENSRNOT00000009233	ENSRNOG00000006859	Insig1	4:2577468–2585691	39.7394	20.5991	−0.947991	4.01374	5.98E−05	0.00982828	0.51835458	
ENSRNOT00000026706	ENSRNOG00000019711	Isoc1	18:54471689–54491596	49.9354	28.9799	−0.78501	3.52998	0.000415584	0.0443191	0.580347809	
ENSRNOT00000015113	ENSRNOG00000043167	Itga9	8:123526903–123837993	8.67236	3.18275	−1.44615	4.45441	8.41E−06	0.00202076	0.366999294	
ENSRNOT00000054983	ENSRNOG00000036703	Itgax	1:187396183–187416231	1.04124	3.27593	1.6536	−3.81958	0.000133681	0.0186697	3.146181476	
ENSRNOT00000049292	ENSRNOG00000001706	Kalrn	11:68195339–68611336	0.548461	0	−1.79769e+308	−1.79769e+308	0.000360543	0.039747	0	
ENSRNOT00000006930	ENSRNOG00000005206	Kcnq3	7:103325195–103364021	0.826586	0.134832	−2.616	4.16018	3.18E−05	0.00593781	0.163119143	
ENSRNOT00000005382	ENSRNOG00000026371	Krt17	10:89185098–89189816	0.339747	2.42074	2.83291	−4.41	1.03E−05	0.00239402	7.125125461	
ENSRNOT00000010660	ENSRNOG00000008057	Krt7	7:140160828–140175532	4.53148	12.9118	1.51064	−4.06718	4.76E−05	0.00823972	2.84935606	
ENSRNOT00000012691	ENSRNOG00000009581	Lce1m	2:186053049–186054252	0.261279	3.64129	3.80078	−4.46855	7.88E−06	0.00190985	13.93640515	
ENSRNOT00000013496	ENSRNOG00000009946	Ldlr	8:20824039–20846920	54.0272	26.6554	−1.01926	4.65674	3.21E−06	0.000888538	0.493370006	
ENSRNOT00000022556	ENSRNOG00000016811	LOC100360880	1:78668540–78673167	7.47652	0.806406	−3.21279	5.58109	2.39E−08	1.24E−05	0.107858469	
ENSRNOT00000000048	ENSRNOG00000000043	LOC100361089	14:1572617–1587520	5.22379	15.9721	1.61238	−3.89499	9.82E−05	0.014564	3.057569313	
ENSRNOT00000040325	ENSRNOG00000021405	LOC100361547	1:244517580–245149649	0.423691	2.12941	2.32937	−3.66948	0.000243041	0.029496	5.025856107	
ENSRNOT00000047694	ENSRNOG00000028826	LOC680161	4:151255240–151413220	2.72431	0.302578	−3.17051	3.87099	0.000108395	0.0157559	0.111065921	
ENSRNOT00000043427	ENSRNOG00000031798	LOC682793	16:10475768–11202166	0	129.85	1.79769e+308	1.79769e+308	2.34E−06	0.000679557	#DIV/0!	
ENSRNOT00000050456	ENSRNOG00000029211	LOC685560	12:20872584–20874637	0.566721	2.99334	2.40104	−3.53543	0.000407118	0.0436082	5.281858269	
ENSRNOT00000000707	ENSRNOG00000000579	Marcks	20:41306445–41309742	29.6393	14.8156	−1.0004	3.60827	0.000308242	0.0352683	0.499863357	
ENSRNOT00000002512	ENSRNOG00000001827	Masp1	11:79532503–79615077	0.68297	2.82874	2.05027	−3.55578	0.000376856	0.0410568	4.141821749	
ENSRNOT00000007577	ENSRNOG00000005695	Mgp	4:173910584–173913947	8.06788	1.58776	−2.3452	3.76003	0.000169895	0.0224737	0.196800151	
ENSRNOT00000038212	ENSRNOG00000025764	Mt1a	19:11261630–11262647	92.2876	34.0727	−1.43752	3.67491	0.000237938	0.0290288	0.369201279	
ENSRNOT00000066331	ENSRNOG00000028016	Ncf2	13:67806515–67834105	1.00012	0	−1.79769e+308	−1.79769e+308	0.000353333	0.0390706	0	
ENSRNOT00000026212	ENSRNOG00000019322	Notch1	3:4631807–4675880	4.55959	8.24001	0.853742	−3.55739	0.000374551	0.0408858	1.807182225	
ENSRNOT00000020532	ENSRNOG00000029792	Ogn	17:20987028–21007525	15.8048	42.463	1.42585	−6.04548	1.49E−09	1.00E−06	2.686715428	
ENSRNOT00000060497	ENSRNOG00000039476	Pcdhb2	18:30104782–30107179	2.25469	0.482476	−2.2244	3.93859	8.20E−05	0.0126831	0.213987732	
ENSRNOT00000011057	ENSRNOG00000008323	Pitpnm3	10:58903761–59017640	0.682975	2.85989	2.06605	−3.79939	0.000145053	0.0198391	4.18740071	
ENSRNOT00000030329	ENSRNOG00000025587	Plagl1	1:7882673–7919508	68.9514	122.108	0.824511	−3.93646	8.27E−05	0.0127749	1.770928509	
ENSRNOT00000016768	ENSRNOG00000011951	Plk2	2:41800744–41806503	34.0069	69.2133	1.02522	−4.82282	1.42E−06	0.00043661	2.035272254	
ENSRNOT00000016991	ENSRNOG00000012495	Podxl	4:58611905–58658598	0.386863	0.0620132	−2.64118	3.70824	0.000208709	0.026187	0.160297573	
ENSRNOT00000015972	ENSRNOG00000011500	Pou2af1	8:54534416–54561348	1.75792	5.39689	1.61826	−3.5917	0.000328529	0.0370255	3.070043005	
ENSRNOT00000016628	ENSRNOG00000012364	Prickle2	4:126571460–126673011	2.17087	0.742499	−1.54781	3.91705	8.96E−05	0.0135983	0.342028311	
ENSRNOT00000021010	ENSRNOG00000015643	Prph	7:137836151–137839931	20.8539	9.35111	−1.15711	4.05215	5.07E−05	0.00866355	0.448410609	
ENSRNOT00000030007	ENSRNOG00000027839	Ptk2b	15:45589212–45718044	20.1607	37.1933	0.883494	−3.88276	0.000103278	0.015227	1.844841697	
ENSRNOT00000052158	ENSRNOG00000008150	RGD1310552	8:79740524–79817585	4.21296	1.10341	−1.93286	3.54479	0.000392919	0.0423988	0.261908492	
ENSRNOT00000051376	ENSRNOG00000018366	RGD1310819	9:36546065–36585558	6.67413	2.12089	−1.65391	3.75686	0.000172062	0.0226632	0.317777748	
ENSRNOT00000022711	ENSRNOG00000016538	RGD1564327	17:86427198–86649274	0.438323	1.97388	2.17097	−4.78272	1.73E−06	0.000521287	4.503254449	
ENSRNOT00000051671	ENSRNOG00000033358	RGD1564380	1:79964554–79966591	3.01913	11.7295	1.95793	−3.97989	6.89E−05	0.0110442	3.885059603	
ENSRNOT00000047522	ENSRNOG00000029141	RGD1564942	5:134778160–134978965	1.09677	0.175426	−2.64432	3.52447	0.000424329	0.0449815	0.159947847	
ENSRNOT00000032690	ENSRNOG00000023814	Rimklb	4:158901834–158936059	33.5082	14.199	−1.23873	3.91219	9.15E−05	0.0138141	0.423747023	
ENSRNOT00000012811	ENSRNOG00000009656	Rspo1	5:144332301–144353646	5.06805	24.8367	2.29297	−6.96689	3.24E−12	3.86E−09	4.900642259	
ENSRNOT00000028510	ENSRNOG00000020992	Selenbp1	2:189840449–189847639	2.66494	7.87451	1.56309	−3.65014	0.000262102	0.0312097	2.954854518	
ENSRNOT00000022202	ENSRNOG00000016512	Sema3b	8:112845710–112852565	8.69307	21.0068	1.27292	−4.64465	3.41E−06	0.000936421	2.41649958	
ENSRNOT00000001916	ENSRNOG00000001414	Serpine1	12:20931995–20942374	157.284	62.8455	−1.32349	6.23914	4.40E−10	3.29E−07	0.399567025	
ENSRNOT00000020043	ENSRNOG00000014870	Slc13a5	10:59133557–59157617	0.621104	0.100966	−2.62097	3.58963	0.000331143	0.0371994	0.162558927	
ENSRNOT00000027234	ENSRNOG00000019996	Slc16a1	2:199860320–199880639	87.5322	147.855	0.756294	−3.58662	0.00033499	0.0375052	1.689149821	
ENSRNOT00000022383	ENSRNOG00000016147	Slc17a6	1:101425974–101466022	12.3913	5.21561	−1.24842	4.49859	6.84E−06	0.00169895	0.420909025	
ENSRNOT00000012683	ENSRNOG00000009480	Slc24a3	3:134018774–134249326	14.6178	27.4457	0.908851	−3.87998	0.000104463	0.0153448	1.877553394	
ENSRNOT00000066904	ENSRNOG00000004928	Sntg2	6:48059245–48263991	1.05054	0.185487	−2.50175	3.62119	0.000293248	0.0340006	0.176563482	
ENSRNOT00000044424	ENSRNOG00000038091	Sohlh2	2:144406047–144428501	0.659867	3.37749	2.3557	−4.51754	6.26E−06	0.00158404	5.118440534	
ENSRNOT00000038572	ENSRNOG00000023551	Sp6	10:85741420–85745602	0.0552052	0.65799	3.57519	−3.58626	0.000335459	0.0375326	11.91898589	
ENSRNOT00000002499	ENSRNOG00000001823	St6gal1	11:79723268–79765638	4.20366	1.53518	−1.45324	3.61795	0.000296947	0.0343149	0.365200801	
ENSRNOT00000007977	ENSRNOG00000006076	Steap2	4:25034175–25053800	13.3337	26.5595	0.994148	−4.18796	2.81E−05	0.00540252	1.991907723	
ENSRNOT00000028675	ENSRNOG00000026951	Susd5	8:118701743–118739369	8.45529	2.62644	−1.68675	4.23269	2.31E−05	0.00460034	0.310626838	
ENSRNOT00000019406	ENSRNOG00000014296	Syt10	7:128115442–128174488	0.159915	1.38967	3.11936	−4.32541	1.52E−05	0.00328267	8.690054091	
ENSRNOT00000027474	ENSRNOG00000020279	Syt11	2:180873774–180897784	21.1451	9.49918	−1.15445	4.33742	1.44E−05	0.00314493	0.449237885	
ENSRNOT00000024030	ENSRNOG00000017628	Tagln	8:48902208–48907693	599.341	1341.02	1.16188	−4.8862	1.03E−06	0.000330673	2.237490844	
ENSRNOT00000055134	ENSRNOG00000026364	Tanc2	10:95145439–95318292	10.3312	5.12396	−1.01168	3.95866	7.54E−05	0.0118549	0.49596949	
ENSRNOT00000027202	ENSRNOG00000020057	Tex101	1:79946338–79949053	5.06684	21.4094	2.07908	−5.08618	3.65E−07	0.000137773	4.225394921	
ENSRNOT00000002867	ENSRNOG00000002093	Tgfbr3	14:3051038–3334311	2.74353	6.72919	1.2944	−3.92853	8.55E−05	0.0130724	2.452748831	
ENSRNOT00000039221	ENSRNOG00000026607	Tnfsf18	13:77136963–77145251	11.558	35.8281	1.63221	−3.95677	7.60E−05	0.0119248	3.099852916	
ENSRNOT00000025606	ENSRNOG00000018943	Tnnc1	16:6639356–6642331	38.374	109.038	1.50663	−5.00048	5.72E−07	0.000199665	2.841455152	
ENSRNOT00000049000	ENSRNOG00000016731	Tpm2	5:59994101–60003261	239.823	427.958	0.835497	−3.59391	0.000325755	0.0368024	1.784474383	
ENSRNOT00000028633	ENSRNOG00000021091	Trank1	8:115701553–115781013	0.208895	0.718386	1.78198	−3.53981	0.000400422	0.0430338	3.438981306	
ENSRNOT00000017892	ENSRNOG00000013053	Trpm6	1:222382666–222502266	1.76725	4.12424	1.22262	−3.84319	0.000121444	0.0172997	2.333704909	
ENSRNOT00000044425	ENSRNOG00000031707	Tuba3a	4:161396176–161405066	1.50508	8.84785	2.55549	−5.44118	5.29E−08	2.54E−05	5.878657613	
ENSRNOT00000052352	ENSRNOG00000032967	Tuba3b	4:183289129–183294677	3.12467	18.4248	2.55987	−6.40278	1.53E−10	1.28E−07	5.896558677	
ENSRNOT00000048874	ENSRNOG00000029071	Unc5c	2:239568541–239721231	0.429189	1.47456	1.7806	−4.09813	4.17E−05	0.00736355	3.435689172	
ENSRNOT00000026559	ENSRNOG00000019598	Vegfa	9:10520729–10536068	3.82035	12.4068	1.69935	−3.81477	0.000136307	0.0189129	3.247555852	
ENSRNOT00000013682	ENSRNOG00000010042	Wdfy2	15:42095911–42222222	0.698329	2.76484	1.98522	−3.49609	0.00047213	0.0488149	3.959222659	

### MMP-7 regulates collagen expression via src, PKA, and ERK1/2

**Figure 4:**
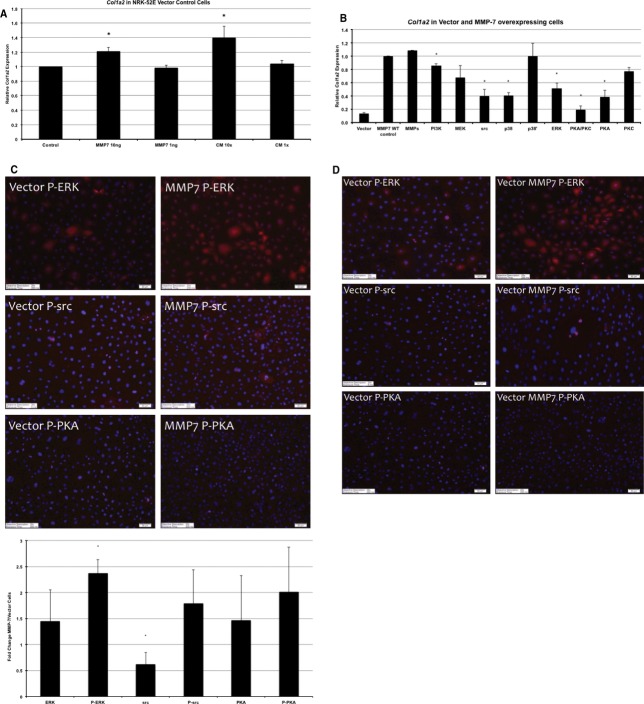
: MMP-7 activates src, PKA, and ERK1/2. (A) *Col1a2* is upregulated in NRK-52E vector control cells after 24-h treatment with exogenous human MMP-7 and conditioned medium (CM) from WT MMP-7 overexpressing cells. *P < 0.05. (B) *Col1a2* upregulation in NRK-52E MMP-7 overexpressing cells is attenuated by inhibition of PI3K (LY294002, 25 μmol\L), src (PP2, 1 μmol\L), p38 (SB203580, 10 μmol\L), ERK1/2 (FR180204, 5 μmol\L), PKA/PKC (Staurosporine, 100 nmol\L), and PKA (KT5720, 1 μmol\L) at 24-h exposure. A second p38 inhibitor (2-(4-Chlorophenyl)-4-(4-fluorophenyl)-5-pyridin-4-yl-1,2-dihydropyrazol-3-one) failed to reproduce the inhibition of SB203508. *P < 0.05. (C) Phosphorylation of ERK, src, and PKA increased in WT MMP-7 overexpressing NRK-52E cells compared to vector control cells as determined by immunofluorescent staining (top panels) and in-cell Western blot (bottom graph). *P < 0.05. (D) Transient (2 h) MMP-7 treatment activates ERK, src, and PKA in vector control NRK-52E cells as determined by immunofluorescent staining for phosphospecific antibodies.

**Figure 5:**
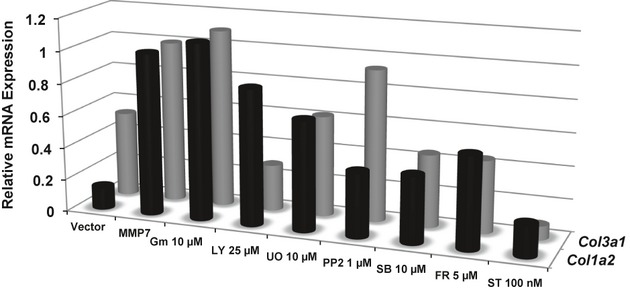
MMP-7 induced up-regulation of Col1a2 and Col3a1 is regulated by distinct pathways as visible by differential responses to selected pathway inhibitors, specifically the PI3K inhibitor LY294002 and the src inhibitor PP2.

Given the importance of collagen overexpression and deposition in chronic kidney dysfunction, we investigated the relationship between MMP-7 and collagen expression, focusing on *Col1a2* regulation, as the overexpression in the MMP-7 cell lines is higher, that is, a fourfold upregulation in the *Col1a2* as compared to twofold in *Col3a1*. Treatment with exogenous MMP-7 as well as conditioned medium from MMP-7 overexpressing cells caused upregulation of *Col1a2* expression in vector control cells (Fig. 4A), further supporting the conclusion that MMP-7 increases collagen expression. To identify a pathway by which MMP-7 upregulates collagen, a range of signaling pathway inhibitors were used. Inhibition of PKA, PKC, PI3K, src, and MEK signaling both via p38 and ERK1/2 abrogated the MMP-7-induced stimulation of *Col1a2* expression (Fig. 4B). Of two p38 inhibitors used, only SB203580 (4-(4-Fluorophenyl)-2-(4-methylsulfinylphenyl)-5-(4-pyridyl)1H-imidazole) abrogated *Col1a2* upregulation, but not the structurally similar 2-(4-Chlorophenyl)-4-(4-fluorophenyl)-5-pyridin-4-yl-1,2-dihydropyrazol-3-one. The PI3K inhibitor LY294002 had a more pronounced effect on *Col3a1* than *Col1a2* suggesting that the two collagens are regulated via different pathways (Fig. 5). Treatment with exogenous MMP-7 has been reported to induce activation by phosphorylation of Akt and ERK1/2 (p44/42 MAPK [mitogen activated protein kinase]) (Varro et al. 2007), as well as epithelial growth factor receptor (EGFR) and MEK (Tan et al. 2005). Increased src, PKA, and ERK1/2 phosphorylation was seen in the MMP-7 overexpressing cells compared to vector controls as assessed by immunofluorescence or in-cell Western blot analysis (Fig. 4C). Importantly, phosphorylation was induced upon treatment with exogenous MMP-7 in vector control cells (Fig. 4D). Taken together, these data suggest that MMP-7 regulates *Col1a2* expression via activation of ERK, p38, PKA, and src pathways.

## Discussion

Chronic kidney disease is accompanied by excessive accumulation of extracellular matrix resulting in renal fibrosis. Fibrosis is a slow and incremental process resulting from repeated injury events accumulating over time. The process, which takes several decades in the human, is accelerated in the rat. As with the individual variability across the human population, the aging process between rat strains varies in respect to the kidney (Baylis and Corman 1998). The male F344 rat used in this study represents a population prone to developing CKD; we detected increased collagen deposition in these animals by 18 months.

MMP-7 [aka matrilysin (Abramson et al. 1995), matrilysin-1, pump – punctuated metalloproteinase (Woessner and Taplin 1988), pump-1 – putative metalloproteinase 1 (Muller et al. 1988; Quantin et al. 1989), matrin (Miyazaki et al. 1990)] is the smallest member of the matrix metalloproteinase family. It is structurally different from other members of the MMP family in that it lacks the C-terminal hemopexin domain, and has instead an atypical sixth exon (Gaire et al. 1994). The protease is synthesized as a 30 kDa (267aa) inactive proform and is then stepwise activated to a final 18 kDa (177aa) form. MMP-7 is fully activated by trypsin and MMP-3, and is partially activated by plasmin, leukocyte elastase (Imai et al. 1995), or aminophenylmercuric acetate (APMA) in vitro. MMP-7 is expressed at very low levels in the adult, and only in a few tissues; however, it has gained attention due to its presence in a variety of disease states including cancer (Ramankulov et al. 2008) and CKD (Musial and Zwolinska 2012). In aging male Fisher 344 rats, MMP-7 was upregulated by over 500-fold in old animals compared to young. MMP-7 activity has been previously reported in association with fibrotic changes in the kidney (Catania et al. 2007) and other fibrotic conditions, such as idiopathic pulmonary fibrosis (Zuo et al. 2002; Rosas et al. 2008) and liver fibrosis (Huang et al. 2005). In these studies, we demonstrate a link between MMP-7 and collagen expression, suggesting a mechanistic link to fibrosis that is counterintuitive given the role of MMP-7 in degradation of the extracellular matrix (Fig. 5).

We found that upregulation of MMP-7 in a normal rat cell-line NRK-52E results in upregulation of two collagen genes, *Col1a2* and *Col3a1*. Both genes are also upregulated in aging Fisher 344 rat kidneys. As *Col1a2* was upregulated fourfold and *Col3a1* only twofold, we focused our inhibitor experiments on type I collagen. In the MMP-7 overexpressing NRK-52E cells, we were able to inhibit the MMP-7-induced upregulation of *Col1a2* by using inhibitors against PKA, PI3K, src, p38, and ERK. When analyzing sequencing data, we were surprised to find no significant changes in expression in any of the major pathway members identified by the inhibitor screen (data not shown). However, it has been reported that inhibiting PI3K and MEK1/2 reversed the proliferative effects of MMP-7 in human gastric myofibroblasts by inhibiting phosphorylation of Akt and ERK1/2 (Varro et al. 2007). Exogenous MMP-7 treatment has also been reported to promote EGFR-activated MEK signaling, as demonstrated by increase in p-EGFR, p-MEK, and p-ERK in pancreatic cancer cells (Tan et al. 2005). We therefore investigated the effect of MMP-7 overexpression on activating phosphorylation status of ERK, src, and PKA. We found increased phosphorylation of each of these proteins in the MMP-7 overexpressing cells compared to vector control cells and we were also able to induce phosphorylation by exogenous MMP-7 treatment of vector control cells.

The human COL1A2 promoter has been described previously (Ramirez et al. 2006). Stimulation of transforming growth factor beta (TGFβ) signaling results in upregulation of Col1A2, via transmembrane serine/threonine kinases and intracellular Smad proteins (Massague et al. 2005). This requires the interactions of Sp1, Smad3/4 (Zhang et al. 2000), and p300/CREB-binding protein (Ghosh et al. 2000) on the COL1A2 promoter. MMP-7 has been implicated in the activation of EGFR and upregulation of TGFβ (Mimori et al. 2004). In the MMP-7 overexpressing cells, however, TGFβ expression was not altered, nor was that of any of the Smad proteins (data not shown). Thus, MMP-7 may be regulating Col1A2 via a non-TGF pathway.

While a paradoxical relationship between expression of MMP-7 and fibrosis has been demonstrated, putatively due to an aberrant wound healing response, (Huang et al. 2005; Wu and Chakravarti 2007; Rodder et al. 2010), a mechanistic link has not been delineated. Our data suggest that MMP-7 increases collagen expression in an autocrine fashion, independent of inflammation. This is consistent with the autocrine activation of ERK1/2 induced by MMP-2 (Xue and Jackson 2008). Our data suggest that the proteolytic activity of MMP-7 may not be required for induction of collagen expression, as the WT MMP-7, which is not processed to an active form in vitro results in elevated *Col1a2* and *Col3a1* expression. The fact that the collagen expression is higher in the WT than in the active mutant could result from the fact that there is significantly more total MMP-7 in the WT that in the active mutant, both at mRNA and secreted protein level. However, the fact that we do not see similar increases in collagen expression in the inactive mutant cell line does suggest a role for activation. Interestingly, in whole kidney lysates from the aging kidney, we have only observed pro-MMP-7 and not the active form, and we have not detected active MMP-7 by zymography in either kidney lysates or urine (data not shown). We conclude, based on the inability to detect active MMP-7 in the aging kidney, that pro-MMP-7 is upregulating collagen expression and, therefore, has a pathophysiological role in renal fibrosis. In addition, MMP-7 has not been reported to degrade Col1a2 and Col3a1. The only collagens demonstrated to be MMP-7 targets are collagen type 4 (Kraft et al. 2001) and collagen type 18 (Lin et al. 2001). However, MMP-7 activates the gelatinases MMP-2 and -9 (von Bredow et al. 1998), and the collagenases MMP-1 and -8, which in turn degrade collagen, but we have not detected MMP-8 expression in the rat kidneys, and MMP-1 expression decreases with age. We have also observed decreased total collagenase and increased gelatinase activity in the aging kidney (24 month) in whole kidney lysates (data not shown). Interestingly this effect is only observed in the presence of APMA to activate latent MMPs. Recent studies have shown that noncatalytic domains of MMPs have signaling effects (Correia et al. 2013; Mori et al. 2013; Vandooren et al. 2013), suggesting that noncatalytic functions of MMPs may have important implications. Although MMP-7 lacks many domains common to other MMPs, future studies will focus on identifying specific MMP-7 domains that mediate collagen overexpression.

In this study we demonstrate a mechanistic link between MMP-7 and fibrosis. The early upregulation of MMP-7 causes increased transcription of *Col1a2* and *Col3a1* genes primarily via PIK3, p38, ERK, src, and PKA signaling, leading to subsequent collagen deposition in the kidney.
